# Persistent Lung Inflammation and Fibrosis in Serum Amyloid P Component (*Apcs^-/-^*) Knockout Mice

**DOI:** 10.1371/journal.pone.0093730

**Published:** 2014-04-02

**Authors:** Darrell Pilling, Richard H. Gomer

**Affiliations:** Department of Biology, Texas A&M University, College Station, Texas, United States of America; University Medical Center Freiburg, Germany

## Abstract

Fibrosing diseases, such as pulmonary fibrosis, cardiac fibrosis, myelofibrosis, liver fibrosis, and renal fibrosis are chronic and debilitating conditions and are an increasing burden for the healthcare system. Fibrosis involves the accumulation and differentiation of many immune cells, including macrophages and fibroblast-like cells called fibrocytes. The plasma protein serum amyloid P component (SAP; also known as pentraxin-2, PTX2) inhibits fibrocyte differentiation *in vitro*, and injections of SAP inhibit fibrosis *in vivo*. SAP also promotes the formation of immuno-regulatory Mreg macrophages. To elucidate the endogenous function of SAP, we used bleomycin aspiration to induce pulmonary inflammation and fibrosis in mice lacking SAP. Compared to wildtype C57BL/6 mice, we find that in *Apcs^-/-^* “SAP knock-out” mice, bleomycin induces a more persistent inflammatory response and increased fibrosis. In both C57BL/6 and *Apcs^-/-^* mice, injections of exogenous SAP reduce the accumulation of inflammatory macrophages and prevent fibrosis. The types of inflammatory cells present in the lungs following bleomycin-aspiration appear similar between C57BL/6 and *Apcs* ^-/-^ mice, suggesting that the initial immune response is normal in the *Apcs^-/-^* mice, and that a key endogenous function of SAP is to promote the resolution of inflammation and fibrosis.

## Introduction

Fibrosing diseases, such as pulmonary fibrosis, cardiac fibrosis associated with hypertensive heart disease, primary myelofibrosis of the bone marrow, liver fibrosis, and renal fibrosis are chronic and debilitating conditions and are an increasing burden for the healthcare system [1]. For instance, pulmonary fibrosis has a survival rate of only 30% five years after diagnosis and an incidence of 1 in 400 in the elderly [2]. There is currently no FDA-approved therapeutic for fibrosis in the US [3]. In fibrosing diseases, scar tissue-like fibrotic lesions cause tissue dysfunction [1], [4]. Fibrosis is mediated by infiltration of blood leukocytes into a tissue, activation and/or appearance of fibroblast-like cells, tissue remodeling, and deposition of extracellular matrix proteins such as collagen [1], [4].

Following their recruitment from the blood into a tissue, monocytes can differentiate into macrophages, dendritic cells, or fibrocytes [4], [5]. Macrophages can have inflammatory M1, pro-fibrotic M2a, or immune-regulatory M2reg characteristics [6], [7]. M1 macrophages produce IL-1β, IL-12, and TNF-α, and modulate host defense against intracellular pathogens, tumor cells, and tissue debris, but are also responsible for tissue damage associated with their release of reactive oxygen species [4], [6]. Pro-fibrotic M2a macrophages are induced by IL-4 or IL-13, are important in the clearance of parasitic infections, and promote tissue remodeling following inflammation, but also secrete a variety of factors that promote fibrosis [1], [8]. Regulatory M2reg macrophages are generally anti-inflammatory, secrete IL-10, and are important during the resolution phase of inflammation [1], [9]. Although these subsets can be viewed as separate, they may be more accurately thought of as a continuum, and may convert from one subset to another depending on the stimuli in an environment [7], [9].

There are multiple sources of fibroblast-like cells present in fibrotic lesions [4]. In addition to the proliferation of resident fibroblasts, proliferation and differentiation of pericytes, and the process of epithelial to mesenchymal transition (EMT), monocytes present within the blood are attracted to sites of injury where they differentiate into spindle-shaped fibroblast-like cells called fibrocytes, which, at least in part, mediate tissue repair and fibrosis [5], [10], [11]. Fibrocytes differentiate from CD14^+^ monocytes [5], [11]–[14]. Fibrocytes have been detected in human pathological conditions including asthma and pulmonary fibrosis [5], [15], , and in animal models of pulmonary fibrosis [5], [17]–[20].

We previously found that the plasma protein Serum Amyloid P component (SAP; PTX2) inhibits human and murine fibrocyte differentiation [12], [21]–[25]. In humans and most mammals, the level of SAP in the plasma is maintained at relatively constant levels, between 20–50 μg/ml [26]. In mice, SAP acts as an acute phase protein, with levels rising up to 20-fold following an inflammatory insult [27], [28]. SAP also inhibits fibrosis, not only by inhibiting fibrocyte differentiation, but also by regulating macrophage polarization [24], [25], [29]–[33]. SAP induces the production of the anti-inflammatory cytokine IL-10, inhibits the formation of pro-fibrotic M2a macrophages, and promotes the formation of immuno-regulatory Mreg macrophages [30], [32]–[35]. SAP is a member of the pentraxin family of proteins that includes C-reactive protein (CRP; PTX1) and pentraxin-3 (PTX3). Injections of human or murine SAP inhibit inflammation and fibrosis in animal models of pulmonary fibrosis [24], [30], [32], cardiac fibrosis [25], [29], dermal wound healing [23], radiation-induced oral mucositis [31], allergic airway disease [34], experimental autoimmune encephalomyelitis [36], and kidney injury [33]. In a Phase 1B clinical trial, injections of recombinant human SAP appeared to improve lung function in pulmonary fibrosis patients [37].

Pentraxins including SAP bind to the receptors for the Fc portion of immunoglobulin G (FcγR) to initiate signaling cascades consistent with FcγR ligation, and SAP has been crystallized bound to FcγRIIa [29], [33], [38]–[42]. Human and murine monocytes express multiple FcγR receptors [43], [44]. In mice, the activating FcγRs are FcγRI, FcγRIII, and FcγRIV, and all three require FcRγ for signaling [43]. The inhibitory receptor is FcγRIIb [45]. SAP appears to act through FcγRI and FcRγ ligation to regulate the differentiation of monocytes into fibrocytes [21]. In addition, the ability of SAP to inhibit fibrosis in mouse models of pulmonary and kidney fibrosis is dependent on the FcRγ component of activating Fcγ receptors [29], [33]. Both human and murine SAP appear to bind to the same receptors [21].

In tissue culture, human and mouse monocytes spontaneously differentiate into fibrocytes when there is no SAP present [12]–[14], [46], [47]. If SAP is the only factor limiting fibrocyte differentiation and promoting immune-regulatory macrophages, this would suggest that mice lacking SAP would have an excess of M2a pro-fibrotic macrophages and fibrocytes, and spontaneously form fibrotic lesions, and thus mice lacking SAP would die quickly. However, *Apcs^-/-^* (Amyloid P component, serum) “SAP knockout mice” appear to be normal and have normal viability [48]–[50], although compared to C57BL/6 mice *Apcs^-/-^* mice are more susceptible to experimental bacterial lung infection [51]. This viability of mice lacking SAP thus suggests that in addition to SAP, other mechanisms inhibit fibrocyte differentiation and regulate fibrosis.

To elucidate the endogenous function of SAP, in this report we used bleomycin to induce inflammation and fibrosis in the lungs of *Apcs^-/-^* mice. We find that the induced lung inflammation and fibrosis in the *Apcs^-/-^* mice is persistent compared to C57BL/6 wild type mice. However, the inflammatory cells present in the lungs after bleomycin aspiration appear similar between C57BL/6 and *Apcs^-/-^* mice, suggesting that the initial response to the insult is independent of SAP, and that a key function of SAP is to promote the resolution of inflammation and fibrosis.

## Methods

### Bleomycin-induced lung inflammation

This study was carried out in strict accordance with the recommendations in the Guide for the Care and Use of Laboratory Animals of the National Institutes of Health. The protocol was approved by the Texas A&M University Animal Use and Care Committee (TAMU AUP #2009-0265 and #2013-0007). All procedures were performed under anesthesia, and all efforts were made to minimize suffering. 4-6 week old C57BL/6 mice (Jackson Laboratory, Bar Harbor, ME) and *Apcs^-/-^* “SAP-knock-out” mice [48] were treated with an oropharyngeal aspiration of 50 μl of 3 U/kg bleomycin (EMD Millipore, Billerica, MA) solution in 0.9% saline or saline alone, as described previously [52]–[54]. 24 hours after bleomycin aspiration, mice were given daily intraperitoneal injections of 50 μg human SAP in 20 mM sodium phosphate buffer or an equal volume of buffer, as described previously [23]–[25], [29], [53]. Arterial oxygen saturation, heart rate, and pulse distention (which measures the local blood flow at the sensor location, and is an indirect indicator of blood flow and blood pressure), were measured using the Mouse Ox vital signs monitor (STARR Life Sciences, Pittsburgh, PA), as described previously [24], [55]. Mice were sedated for 60 seconds with 4% isoflurane in oxygen, removed to room air, and then parameters monitored until the mice were active. To measure organ weights, following euthanasia mice were weighed, blood was collected from the abdominal aorta to isolate serum, and the heart, spleen, kidneys, lungs, and liver were removed and weighed. Mice were euthanized at 7 or 21 days after bleomycin aspiration, weighed, blood was collected from the abdominal aorta to isolate serum, and the lungs were then perfused through the trachea with 300 μl of PBS three times to collect cells by bronchoalveolar lavage (BAL) as described previously [53], [54], [56]. The BAL cells were collected by centrifugation at 300×g for 5 minutes, and the supernatants were transferred to eppendorf tubes, flash frozen with liquid nitrogen, and stored at −80°C. The cells collected from BAL were resuspended in 4% BSA-PBS, counted with a hemocytometer, and cytopsins prepared as described previously [53], [54], [57]. After BAL, lungs were inflated with pre-warmed optimal cutting temperature (OCT) compound (VWR, Radnor, PA), embedded in OCT, frozen on dry ice, and stored at −80°C as described previously [24], [53], [54].

### Fibrocyte differentiation assay

Mouse spleen cells were isolated as described previously, except that the spleens were digested with Accutase (Global Cell Solutions, Charlottesville, VA) for 30 minutes at room temperature according to the manufacturers' instructions [21], [22]. Spleen cells were cultured in FibroLife (Lifeline Cell Technology, Frederick, MD) serum-free medium (SFM) including 50 ng/ml murine IL-13 and 25 ng/ml murine M-CSF (PeproTech, Rocky Hill, NJ), as described previously [21], [22], [46]. Spleen cells were cultured in flat-bottomed, 96-well, tissue-culture plates at 3.5×10^5^ cells/well in 200 μl with human SAP at the indicated concentrations. On day 3 of the incubation, wells were supplemented with IL-13 and M-CSF, as described previously [21], [22]. After 5 days, plates were air-dried, fixed with methanol, and stained with eosin and methylene blue, as described previously [12]–[14], [21], [22], [46]. Fibrocytes were counted using the following criteria: an adherent cell with elongated spindle-shaped morphology and an oval nucleus, as described previously [12]–[14], [21], [22], [46]. IC50 values were obtained by fitting a sigmoidal dose response curve to the data.

### Flow Cytometry

Spleen cells were analyzed by flow cytometry, as described previously [21], [22], [46]. Cells were stained using 5 μg/ml of antibodies against CD3 (clone 17A2, rat IgG2b, BD Biosciences, San Jose, CA) to detect T cells; CD11b (clone M1/70, rat IgG2b, BioLegend, San Diego, CA) to detect neutrophils, monocytes, and macrophages; CD11c (clone 223H7, rat IgG2a, MBL International, Woburn, MA) to detect dendritic cells and macrophages; CD16/32 to detect the epitope common to FcγRII and FcγRIII (clone 93, rat IgG2a, BioLegend), CD45R/B220 (clone RA3-6B2, rat IgG2a, BD Biosciences) to detect B cells; Ly6G (clone 1A8, rat IgG2a, BD Biosciences) to detect neutrophils; GR-1 (clone RB6-8C5, rat IgG2b, BD Biosciences) to detect Ly-6C and Ly-6G macrophage subsets and neutrophils; and isotype-matched irrelevant rat monoclonal antibodies (BioLegend). The secondary antibody was a FITC-conjugated mouse F(ab')_2_ anti-rat IgG (Jackson ImmunoResearch, West Grove, PA) used at 2.5 μg/ml. After blocking with PBS/20% rat serum (Sigma-Aldrich, St. Louis, MO) for 30 minutes at 4°C, cells were incubated with 2.5 μg/ml biotinylated anti-CD45 (clone 30-F11, rat IgG2b, BioLegend). After a 30 minute incubation on ice, cells were washed and then incubated with streptavidin-APC (BioLegend). After a 30-minute incubation on ice, cells were washed twice, resuspended in 100 μl ice-cold PBS/2% BSA, and analyzed using a C6 flow cytometer (Accuri, Ann Arbor, MI).

### Immunochemistry

BAL cytospins and frozen lung tissue sections (5 μm) were prepared and immunochemistry performed as described previously [24], [35], [53], [54]. Antibodies to Ly6G (BioLegend) were used to detect neutrophils, CD11b (BioLegend) to detect blood and inflammatory macrophages, CD11c (MBL International) to detect resident lung macrophages and dendritic cells, Gr-1 (BioLegend) to detect infiltrating macrophages and neutrophils, CD206 (BioLegend) to detect the mannose receptor on macrophages and dendritic cells, and CD45 (BioLegend) to detect all leukocytes. Isotype-matched mouse irrelevant antibodies were used as controls. Lung sections were also stained with H&E or sirius red to detect inflammation, fibrosis, and collagen, as described previously [24], [35], [53], [54]. Several studies have shown that histological analysis of fibrosis and collagen content by techniques such as sirius red staining, correlates well with total collagen content as measured by hyproxyproline or sirius red (Sircol) assays [58]–[62].

### Lung collagen content assays and BAL cytokine measurements

Lungs of naïve mice were purged of blood, excised, weighed, snap-frozen in liquid nitrogen, and stored at −80°C, as described previously [24]. Lungs were digested with 0.1 mg/ml pepsin in 0.5 M acetic acid overnight at 4°C. Tissue debris was removed by centrifugation, and collagen determined using the sirius red dye assay, as described previously [24], [52], [63]. Primary BAL fluids were assessed for IL-13 by ELISA (Peprotech), following the manufacturer's instructions. Total protein in the primary BAL was assessed by absorbance at 260 nm, 280 nm and 320 nm, using a Synergy MX spectrophotometer (BioTek, Winooski, VT). In addition, BAL fluids were analyzed by PAGE, and gels stained with Coomassie Brilliant Blue, as described previously [12], [24].

### Measurement of endogenous murine SAP and exogenous human SAP

Blood from untreated mice, and mice euthanized at day 7 and 21 after bleomycin aspiration was collected and serum stored at −20°C. Exogenous human SAP was measured by ELISA, as described previously [12], [64]. This ELISA does not cross-react with murine SAP or other murine serum proteins (data not shown). To detect endogenous murine SAP, serum was analyzed by western blotting using sheep anti-mouse SAP antibodies (R&D Systems, Minneapolis, MN), as described previously [12], [21], [24], [64].

### Statistics

Statistical analysis was performed using Prism (GraphPad Software, La Jolla, CA). Statistical significance between two groups was determined by t test, or between multiple groups using analysis of variance (ANOVA), and significance was defined as p<0.05.

## Results

### Naïve *Apcs^-/-^* mice have altered lung function

**Figure 1:**
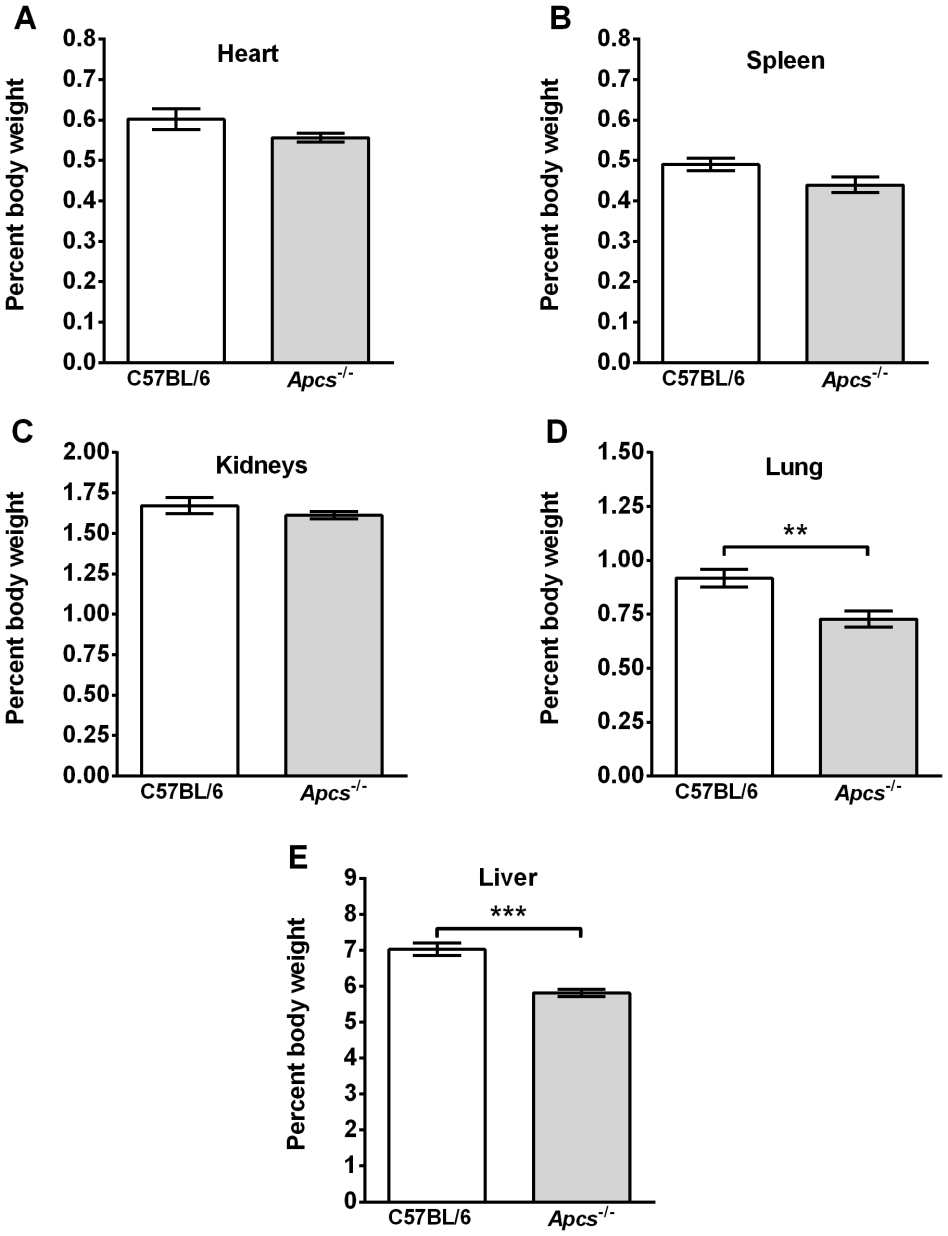
Organ weights in *Apcs^-/-^* mice. Body and organ weights from animals not exposed to bleomycin or exogenous SAP were measured. Organ weights are expressed as a percent of body weight. **A**) heart, **B**) spleen, **C**) kidneys, **D**) lungs, and **E**) liver. Values are mean ± SEM (n = 12 for C57BL/6; n = 24 for *Apcs^-/-^*). **p<0.01, ***p<0.001 (t-test).

**Figure 2:**
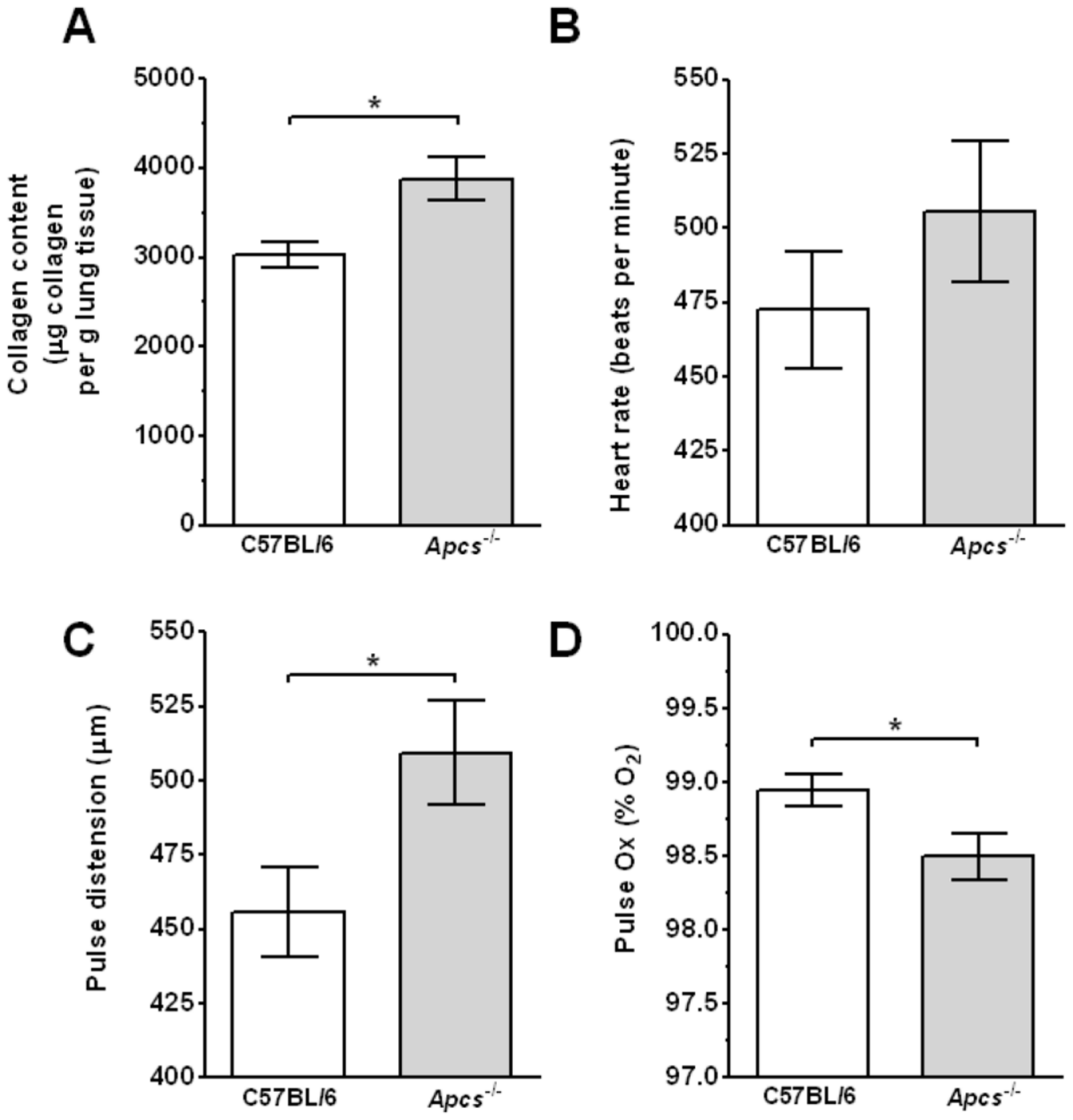
*Apcs^-/-^* mice have increased lung collagen and reduced lung function. **A**) Lungs were assessed for collagen content. Values are mean ± SEM (n = 8 per group). Mice were assessed for **B**) heart rate, **C**) pulse distension, and **D**) peripheral blood oxygen content (pulse Ox). Values are mean ± SEM (n = 21 for C57BL/6; n = 14 for *Apcs^-/-^*). *p<0.05 (t-test).

*Apcs^-/-^* mice were originally generated on a 129 background and then backcrossed to C57BL/6 mice [48], [65]. The back-crossed F2 *Apcs^-/-^* mice develop glomerulonephritis in adults >6 months old. This hybrid genetic background (129×C57BL/6) has since been shown to generate autoantibodies and glomerulonephritis independent of the *Apcs^-/-^* locus [66]–[68]. However, the mice used in the experiments described below were backcrossed for 10 generations to C57BL/6 mice as described previously [66], [67], [69]. In addition, after transfer from the Botto lab, the *Apcs^-/-^* mice were re-derived and then backcrossed for an additional two generations to C57BL/6 mice. Mice were checked for *Apcs^-/-^* gene deletion by PCR, and the sera were screened for absence of SAP protein by ELISA and/or western blotting (data not shown). To further reduce the possible effects of autoimmunity found in >6 months old mice, all experiments were performed on 4–6 week old mice. However, as young *Apcs^-/-^* mice may have altered lung and kidney characteristics due to the presence of autoantibodies, low grade fibrosis, and/or underlying infections, we compared the organ weights of 4–6 week old naïve C57BL/6 and *Apcs^-/-^* mice, using organ weight increase as an indicator of edema associated with inflammation [70]. The heart, spleen, and kidney weights (as a percent of body weight) were similar between *Apcs^-/-^* and C57BL/6 mice, but the liver and lung weights were lower in *Apcs^-/-^* mice (Figure 1). Compared to C57BL/6 mice, the lungs of *Apcs^-/-^* mice had increased collagen content (Figure 2A), although the lungs appeared histologically normal (data not shown). However, we could not detect the presence of the pro-fibrotic cytokine IL-13 (detection limit 7.8 pg/ml) in BAL samples (data not shown). There was a significant difference in the pulse oximetry and pulse distention (which measures the local blood flow at the sensor location and is an indirect indicator of blood flow and blood pressure), but not heart rate, between naïve C57BL/6 and *Apcs^-/-^* mice (Figure 2B–D). Together, these observations suggest that in mice, SAP slightly increases lung and liver mass, decreases lung collagen content, and potentiates lung function.

### *Apcs^-/-^* mice have a reduced number of spleen monocytes and increased inhibition of fibrocyte differentiation by SAP

**Figure 3:**
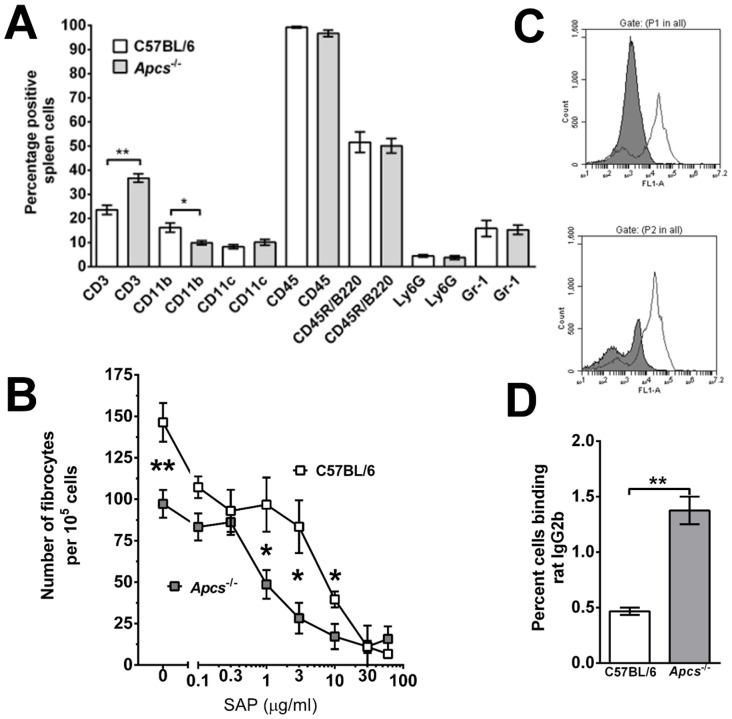
Spleen leukocyte populations and fibrocyte differentiation in *Apcs^-/-^* mice. **A**) Spleen cells were analyzed for leukocyte cell populations using antibodies and flow cytometry. Values are mean ± SEM (n = 3 for C57BL/6; n = 4 for *Apcs^-/-^* mice). *p<0.05, **p<0.01 (t-test). **B**) Spleen cells were cultured for 5 days in the presence or absence of the indicated concentrations of SAP. After 5 days, cells were air-dried, fixed, and stained, and the number of fibrocytes was counted. Values are mean ± SEM (n = 3 for C57BL/6; n = 4 for *Apcs^-/-^* mice). *p<0.05 (t-test). **C**) Spleen cells from C57BL/6 (top panel) and *Apcs^-/-^* (bottom panel) mice were analyzed by flow cytometry for CD45R/B220 (white histogram) and CD16/32 (grey histogram) using a mAb (clone 93) to a common epitope of FcγRII and FcγRIII. Flow cytometry data is a representative of four independent experiments. **D**) Spleen cells were incubated with rat IgG2b antibodies and IgG2b binding was analyzed by flow cytometry. Values are mean ± SEM (n = 3 for C57BL/6; n = 4 for *Apcs^-/-^* mice). **p<0.01 (t-test).

As the subsequent experiments aim to investigate the role of SAP in bleomycin-induced lung inflammation and fibrosis, we tested if *Apcs^-/-^* mice had altered immune cell numbers or an altered response to SAP. We found no significant difference in the number of cells recovered from the spleens of C57BL/6 (90±5×10^6^; mean ± SEM; n = 3) and *Apcs^-/-^* mice (100±6×10^6^; n = 4). Compared to C57BL/6 mice, *Apcs^-/-^* mice have increased numbers of CD3+ T cells and reduced numbers of CD11b+ monocytes and macrophages in the spleen (Figure 3A). We found no significant difference in the percentage of CD11c+ macrophages and dendritic cells, CD45+ leukocytes, CD45R/B220+ B cells, Ly6G+ neutrophils, or Gr-1+ inflammatory macrophages (Figure 3A). Spleen monocytes are CD11b+, CD11c−, Gr-1-, whereas spleen macrophages are CD11b+, CD11c+, and can be either Gr-1 positive or negative [71]–[73]. As the percentage of CD11c, Ly6G, and Gr-1 positive spleen cells was not different between C57BL/6 and *Apcs^-/-^* mice, these data suggest that the lower numbers of CD11b+ cells may reflect a lower number of spleen monocytes.

Spleen cells from C57BL/6 and *Apcs^-/-^* mice were cultured in SFM for 5 days in the presence or absence of human SAP. Compared to the C57BL/6 spleen cells, in the absence of exogenous SAP, there were fewer fibrocytes in cultures of *Apcs^-/-^* spleen cells (Figure 3B). The decreased percentage of CD11b+spleen cells in the *Apcs^-/-^* mice (Figure 3A), and the decreased number of fibrocytes that differentiate from a given number of *Apcs^-/-^* spleen cells (Figure 3B), suggests that there is a decreased number of spleen monocytes that are capable of differentiating into fibrocytes in *Apcs^-/-^* mice.

We also tested if *Apcs^-/-^* mice had an altered fibrocyte response to SAP. As previously observed [21], [22], SAP inhibited the differentiation of fibrocytes from C57BL/6 spleen cells with an IC_50_ of 4.7±2.7 μg/ml (mean ± SEM) (Figure 3B), whereas *Apcs^-/-^* spleen cells had an IC_50_ of 0.9±0.1 μg/ml. These data suggest that the presence of endogenous SAP desensitizes monocytes to exogenous SAP, possibly by downregulating SAP receptor expression and/or signal transduction pathways. To determine if the absence of endogenous SAP alters FcγR expression, we stained spleen cells for FcγRs. We found no significant difference in the number of FcγRI positive cells, or their staining intensity (data not shown). However, *Apcs^-/-^* spleen cells had two discrete populations of spleen cells staining for a common epitope of FcγRII and FcγRIII, whereas spleen cells from C57BL/6 contain a single population (Figure 3C). Rat IgG2b binds to FcγR with high affinity [74], [75]. Compared to C57BL/6 spleen cells, *Apcs^-/-^* mice had increased numbers of rat IgG2b-binding spleen cells (Figure 3D). These data suggest that endogenous SAP either alters the expression of FcγR, or binds to the FcγR to prevent antibody binding.

### SAP is required to maintain pulse distension at 21 days after bleomycin treatment

**Figure 4:**
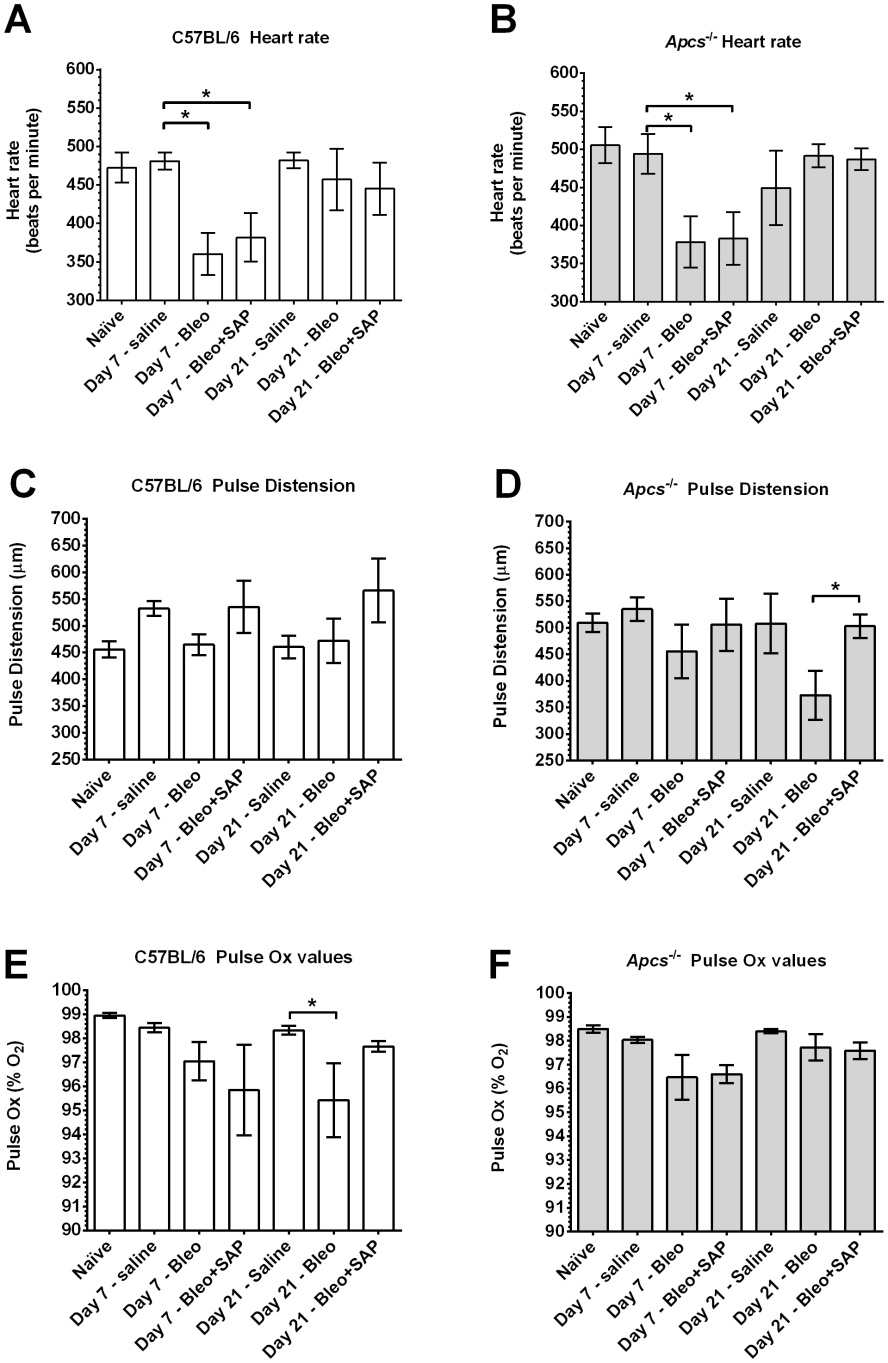
Heart rate, pulse distension, and pulse Ox after bleomycin aspiration. At day 7 and 21 after bleomycin aspiration, C57BL/6 (**A, C, E**) and *Apcs^-/-^* (**B, D, and F**) mice were assessed for **A**) and **B**) heart rate, **C**) and **D**) pulse distension and **E**) and **F**) pulse Ox. Values are mean ± SEM (n = 4–9 mice per time point). *p<0.05 (1-way ANOVA, Tukey's test).

Bleomycin aspiration leads to epithelial cell damage, inflammation, collagen deposition, and fibrosis [76], [77]. These changes also reduce lung function and peripheral blood oxygen content [24], [78]. To determine if *Apcs^-/-^* mice had an altered response to bleomycin aspiration, we measured heart rate, pulse distension, and arterial oxygen saturation (pulse Ox) at 7 days after aspiration, when there is acute inflammation and alveolar cell death, and at 21 days when there is peak fibrosis and the acute toxic effects of bleomycin have receded [76]. We observed that at day 7 in both C57BL/6 and *Apcs^-/-^* mice, bleomycin caused a significant reduction in heart rate compared to mice receiving saline (Figures 4A and 4B). This effect was present in mice treated with either SAP injections or buffer. At 21 days after bleomycin aspiration, the heart rate appeared normal in C57BL/6 and *Apcs^-/-^* mice irrespective of SAP treatment (Figures 4A and 4B). There appeared to be no significant changes in pulse distension in C57BL/6 mice (Figure 4C). At 21 days after bleomycin aspiration, *Apcs^-/-^* mice had a significant reduction in pulse distension compared to *Apcs^-/-^* mice treated with SAP (Figure 4D). At 21 days after bleomycin aspiration, there appeared to be a reduction in arterial blood oxygen saturation in C57BL/6 mice (Figure 4E). However, in mice treated with SAP, there was no significant change in arterial blood oxygen saturation (Figure 4E). Together, these results indicate that 21 days after bleomycin treatment, *Apcs^-/-^* mice show an abnormally low pulse distension, and that this phenotype can be rescued by SAP injections.

### *Apcs^-/-^* mice have increased numbers of inflammatory macrophages following aspiration of bleomycin

**Figure 5:**
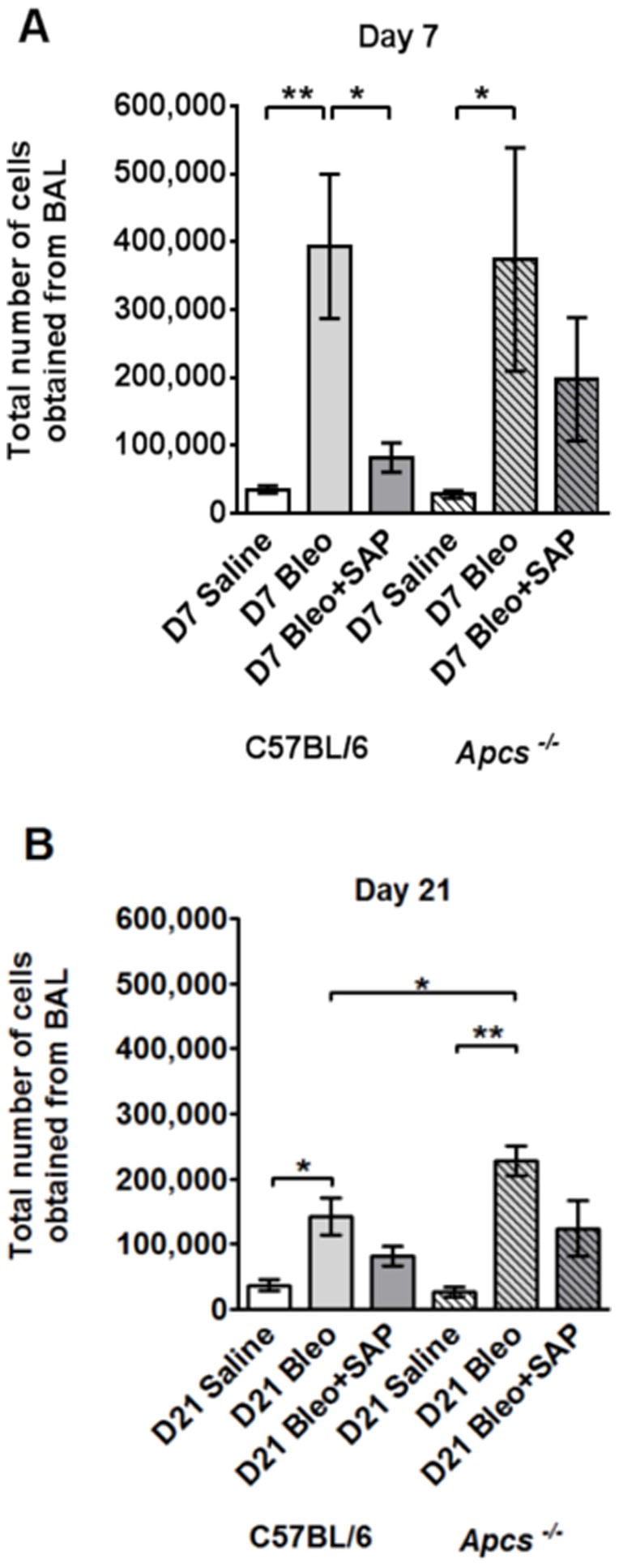
Changes in BAL cell number following bleomycin aspiration. The total number of cells collected from the BAL is shown in **A**) for day 7 and **B**) for day 21. Values are mean ± SEM (n = 4–6 mice per group). *p<0.05, **p<0.01 (by ANOVA between groups, and t-test when comparing C57BL/6 and *Apcs^-/-^*).

SAP injections inhibit neutrophil, macrophage, and fibrocyte accumulation in the lungs of bleomycin-treated C57BL/6 mice [24], [30], [53]. We thus tested the hypothesis that bleomycin aspiration would cause increased numbers of cells in the lungs, and/or a more persistent inflammatory and fibrotic response, in *Apcs^-/-^* mice. Following bleomycin aspiration, C57BL/6 and *Apcs^-/-^* mice were treated with daily intraperitoneal injections of SAP or buffer, and then euthanized at 7 days (to measure the inflammatory response) and 21 days (to measure the fibrotic response) [76], [79]. The weakly adhered cells from the airways were collected by bronchoalveolar lavage (BAL), counted, and cell morphology was used to determine the total number of neutrophils, macrophages, and lymphocytes in the BAL. At day 7, the BAL from mice receiving bleomycin alone contained significantly more cells than the saline control for both genotypes, and the mice given bleomycin and then treated with SAP tended to have less cells than the mice given bleomycin (Figure 5A). These data suggest that the early response to bleomycin-induced inflammation is similar in C57BL/6 and *Apcs^-/-^* mice.

At day 21, the BAL from C57BL/6 and *Apcs^-/-^* mice given bleomycin aspiration without SAP treatment contained significantly more cells than the saline control (Figure 5B). However, bleomycin caused significantly more cells to appear in the BAL of *Apcs^-/-^* mice compared to C57BL/6 mice (Figure 5B). There was no significant difference in the number of cells in the BAL of C57BL/6 and *Apcs^-/-^* mice given saline alone, or given bleomycin and then treated with SAP (Figure 5B). These data suggest that a phenotype of *Apcs^-/-^* mice is that they have a more persistent inflammatory response compared to C57BL/6 mice, and that this effect can be decreased by SAP injections.

**Figure 6:**
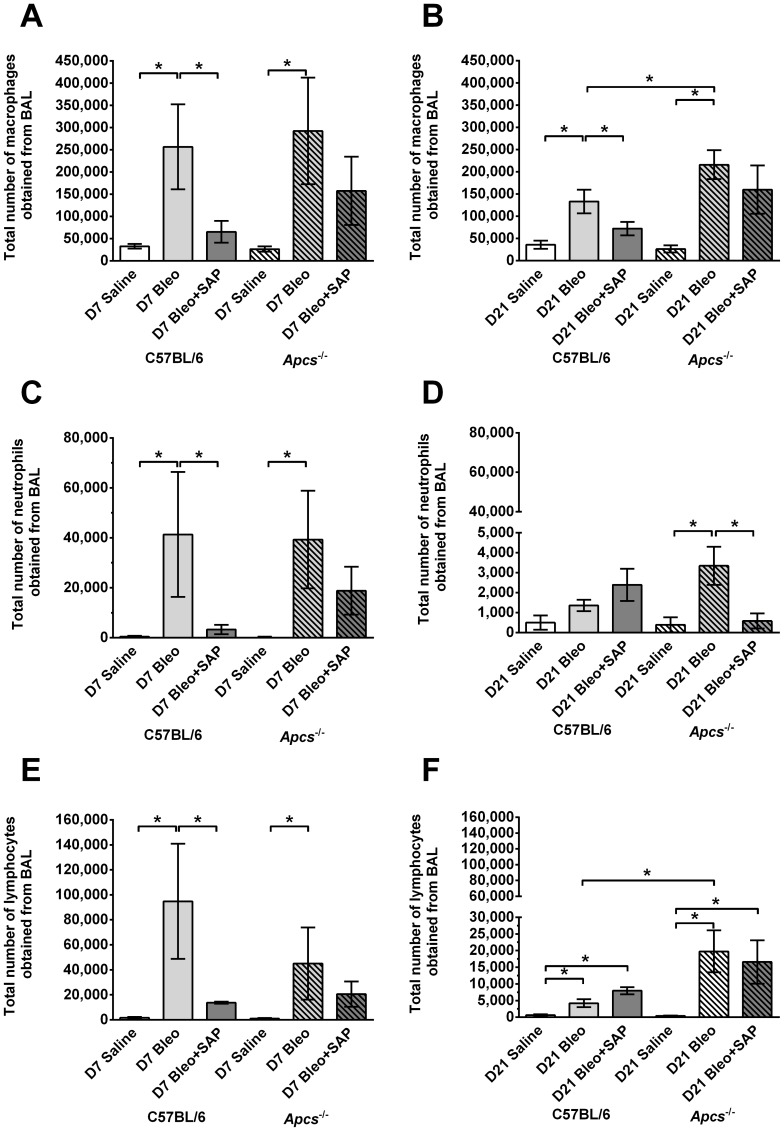
Changes in BAL leukocyte populations following bleomycin aspiration. Graphs show the total number of **A**) and **B**) macrophages, **C**) and **D**) neutrophils, and **E**) and **F**) lymphocytes obtained in the BAL at day 7 (**A**, **C**, and **E**) and day 21 (**B**, **D**, and **F**). Values are mean ± SEM (n = 4–6 mice per group). *p<0.05 (by ANOVA between groups, and t-test when comparing C57BL/6 and *Apcs^-/-^*).

Cell morphology was used to determine the total number of macrophages, lymphocytes, and neutrophils in the BAL. At day 7, compared to saline aspiration, there were elevated numbers of macrophages, neutrophils, and lymphocytes in the BAL of both C57BL/6 and *Apcs^-/-^* mice given bleomycin, and these numbers were reduced by SAP treatment (Figure 6A, B, and C). At day 21, compared to C57BL/6 mice, there were significantly more macrophages and lymphocytes in the BAL of *Apcs^-/-^* mice following bleomycin aspiration, and these numbers were reduced somewhat by SAP treatment (Figure 6B and F).

**Figure 7:**
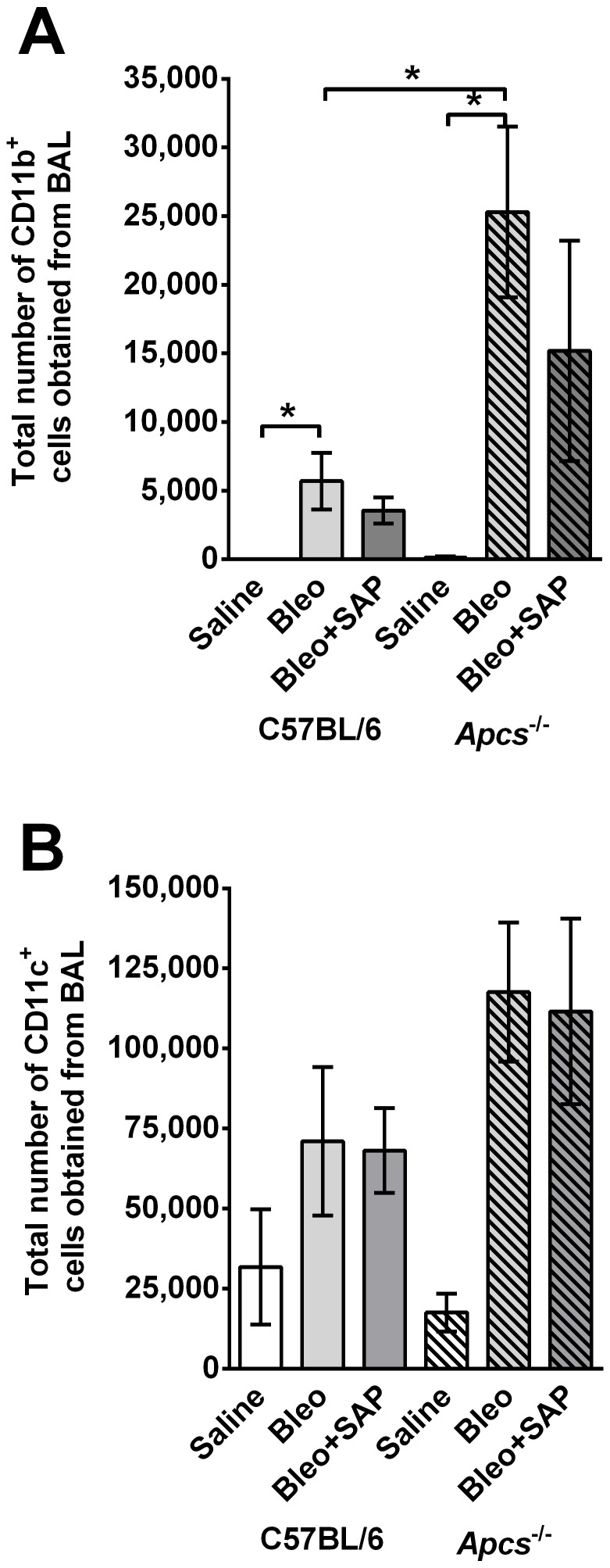
Changes in BAL CD11b^+^ inflammatory cells and CD11c^+^ resident lung cells in C57BL/6 and *Apcs^-/-^* mice. Graphs show the total number of **A**) CD11b positive and **B**) CD11c positive cells collected from the BAL at day 21. Values are mean ± SEM (n = 3–6 per group). *p<0.05, (t-test).

BAL cells were also labeled with antibodies to identify resident alveolar macrophages (CD11b- CD11c+) or newly recruited inflammatory macrophages (CD11b+ CD11c+) [80]–[83]. At 21 days post-bleomycin aspiration, compared to C57BL/6 mice, there were significantly more CD11b+ cells in the BAL of *Apcs^-/-^* (Figure 7A). SAP treatment slightly reduced these numbers in both C57BL/6 and *Apcs^-/-^* mice. There was no significant difference in the number of resident alveolar CD11c+ cells between C57BL/6 and *Apcs^-/-^* mice that aspirated bleomycin (Figure 7B). These data indicate that SAP limits CD11b+ inflammatory macrophage accumulation in mouse lungs at 21 days after bleomycin aspiration.

**Figure 8:**
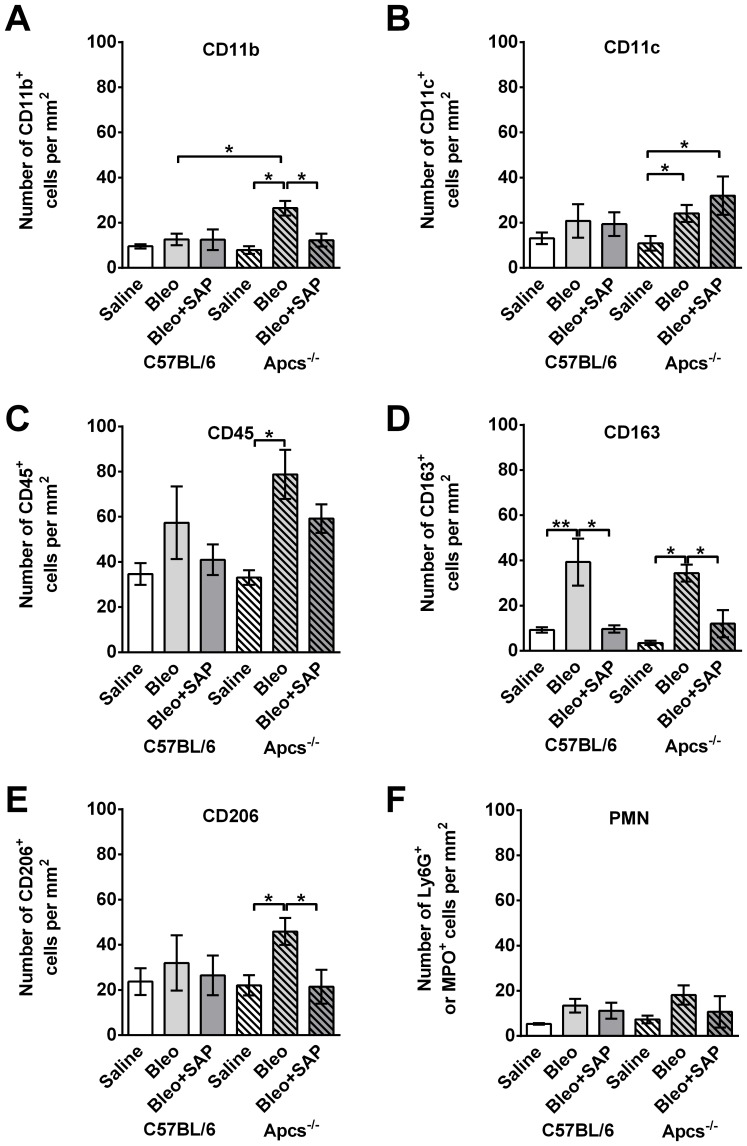
Changes in tissue leukocyte population in C57BL/6 and *Apcs^-/-^* mice following bleomycin instillation. Cryosections of mouse lungs were labeled with antibodies against **A**) CD11b (neutrophils and inflammatory macrophages), **B**) CD11c (resident macrophages and dendritic cells), **C**) CD45 (total leukocytes), **D**) CD163 (macrophages), **E**) CD206 (macrophages), and **F**) Ly6G (neutrophils). Values are mean ± SEM (n = 4–6 mice per group). Significance was determined by ANOVA within treatment groups and t-test when comparing C57BL/6 and *Apcs^-/-^* mice.

Following BAL, lungs were sectioned and stained with antibodies to detect cells that were not removed by BAL. At 21 days after bleomycin aspiration, compared to C57BL/6 mice, there were significantly more CD11b+ cells in the lungs of *Apcs^-/-^* mice (Figure 8A). In addition, SAP treatment significantly reduced the number of CD11b+ cells in the lungs of *Apcs^-/-^* mice (Figure 8A). At day 21, bleomycin also cause an increase in CD11c+, CD45+, CD163+, and CD206+ cells in *Apcs^-/-^* lungs, and SAP injections reduced the number of CD163+ and CD206+ cells (Figure 8B, C, D, and E). In C57BL/6 mice, following bleomycin aspiration, the only significant difference compared to the saline control was an increase in CD163+ cells, which was also reduced with SAP injections (Figure 8D). There was no difference in the numbers of CD11b, CD11c, CD45, or CD206 positive cells in the lungs of C57BL/6 mice treated with bleomycin compared with those treated with bleomycin and then receiving SAP injections (Figure 8). There were no significant changes in the numbers of neutrophils in either C57BL/6 or *Apcs^-/-^* mice (Figure 8F). These results suggest that a phenotype of *Apcs^-/-^* mice is an abnormally high number of CD11b inflammatory macrophages present in the lungs after BAL at 21 days after bleomycin treatment, and that this phenotype is rescued by SAP injections.

### Bleomycin aspiration does not increase endogenous SAP levels

**Figure 9:**
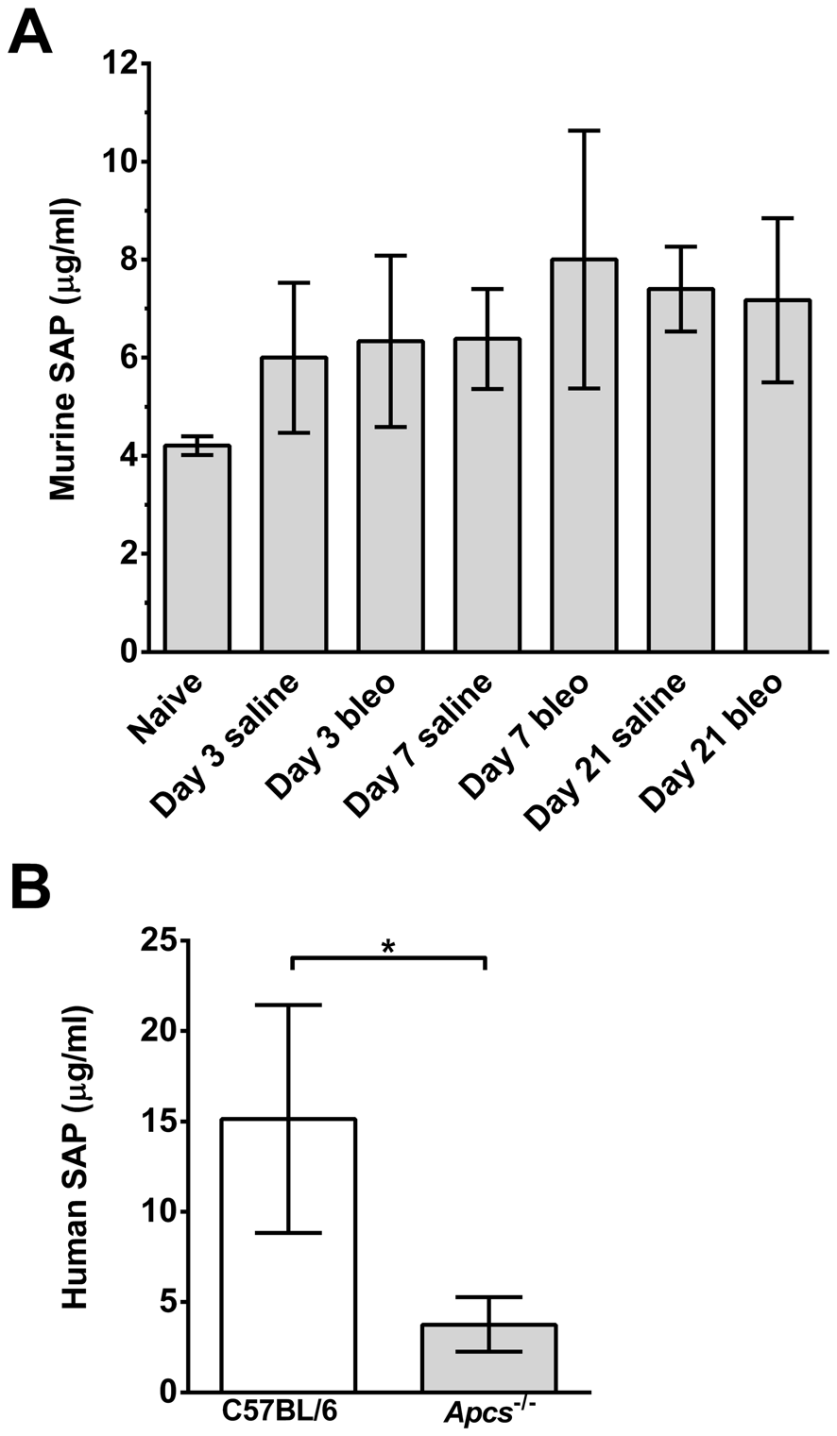
Endogenous and exogenous SAP levels in C57BL/6 and *Apcs^-/-^* mice. **A**) Serum was collected at day 0, 3, 7, and 21 from C57BL/6 mice that had received saline or bleomycin. Serum was assayed for the presence of endogenous murine SAP by western blotting. Values are mean ± SEM (n = 3–7 mice per group). **B**) C57BL/6 and *Apcs^-/-^* mice were injected with human SAP for 6 days following bleomycin aspiration. Serum was collected at day 7 and human SAP levels determined by ELISA. Values are mean ± SEM (n = 4–6 mice per group).

Exogenous SAP injections reduce inflammation and fibrosis in many organ systems [3], [84]. However, as SAP is an acute phase response protein in mice [85], [86], the stimuli that induce inflammation and fibrosis may elevate endogenous SAP. Therefore, we tested if bleomycin aspiration increased endogenous SAP levels in the serum of C57BL/6 mice. We did not observe a significant increase in murine serum SAP at 3, 7, or 21 days after bleomycin aspiration (Figure 9A). These data indicate that bleomycin aspiration does not appear to promote a persistent systemic increase in SAP levels. As the lack of endogenous SAP in the *Apcs^-/-^* mice may alter the pharmacokinetics for exogenous SAP, we collected serum from C57BL/6 and *Apcs^-/-^* mice injected daily for 6 days with 50 μg SAP following bleomycin aspiration. We observed that exogenous human SAP was detectable at day 7 in the sera of both C57BL/6 and *Apcs^-/-^* mice (Figure 9B), although the levels were lower in *Apcs^-/-^* mice (Figure 9B). In addition, we did not detect any compensatory changes in the levels of serum CRP between C57BL/6 and Apcs^-/-^ mice either at baseline or at 7 or 21 days after bleomycin challenge (data not shown).

### Endogenous or exogenous SAP has no effect on bleomycin-induced lung damage

**Figure 10:**
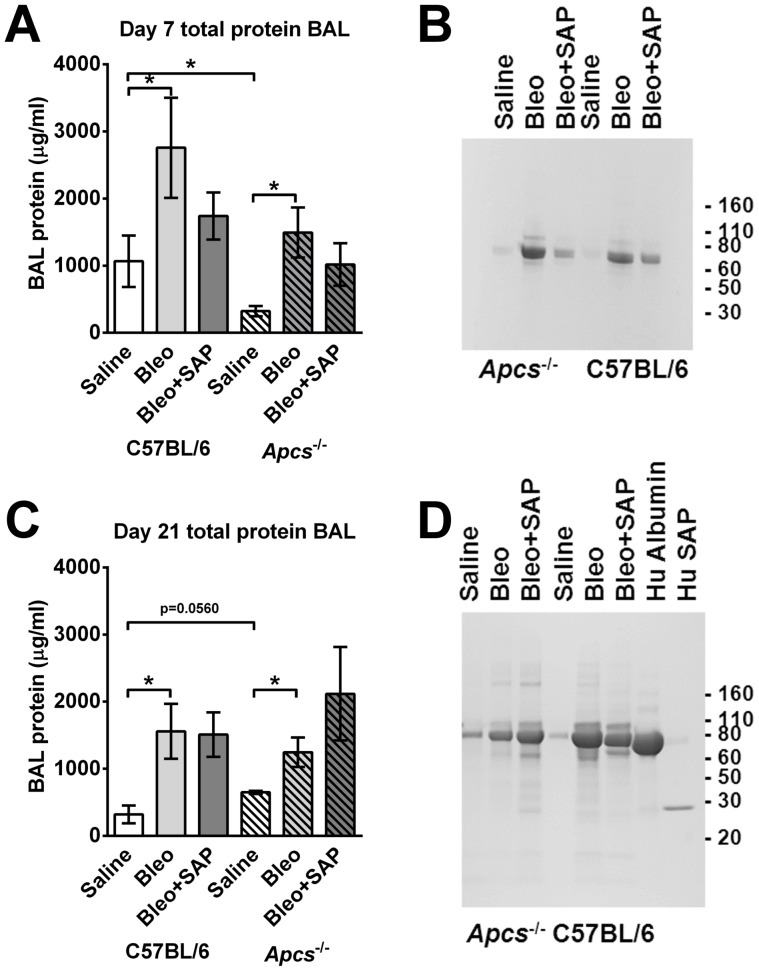
SAP has no effect on bleomycin-induced lung damage. BAL was collected at day 7 (**A** and **B**) and day 21 (**C** and **D**) following saline or bleomycin instillation, and SAP injections. A and C) Total protein was assessed by spectrophotometry. Values are mean ± SEM (n = 3 for C57BL/6; n = 5 for *Apcs^-/-^*). * indicates p<0.05 (t test). B and D) BAL were analyzed by PAGE on a 4–15% reducing gel, and stained with Coomassie. In D, BAL samples were analyzed alongside human albumin and human SAP. The gel is representative of three independent experiments.

To determine if exogenous or endogenous SAP levels could also regulate bleomycin-induced lung damage, we measured total lung BAL protein levels, as a measure of lung tissue damage following bleomycin aspiration [87], [88]. We found that at day 7, bleomycin caused an increase in BAL protein levels, and that *Apcs^-/-^* mice had lower protein levels compared to C57BL/6 mice (Figure 10A). The bleomycin-induced BAL protein appeared to be mainly albumin, as determined by PAGE (Figure 9B and 9D). SAP injections did not significantly affect the protein levels present in the BAL. At day 21, following bleomycin aspiration BAL protein levels were also increased in the C57BL/6 and *Apcs^-/-^* mice (Figure 10C and 10D). SAP injections had no significant effect on BAL protein levels. These data suggest that SAP does not regulate edema or epithelial barrier destruction following bleomycin instillation.

### *Apcs^-/-^* mice have increased fibrotic response following aspiration of bleomycin

**Figure 11:**
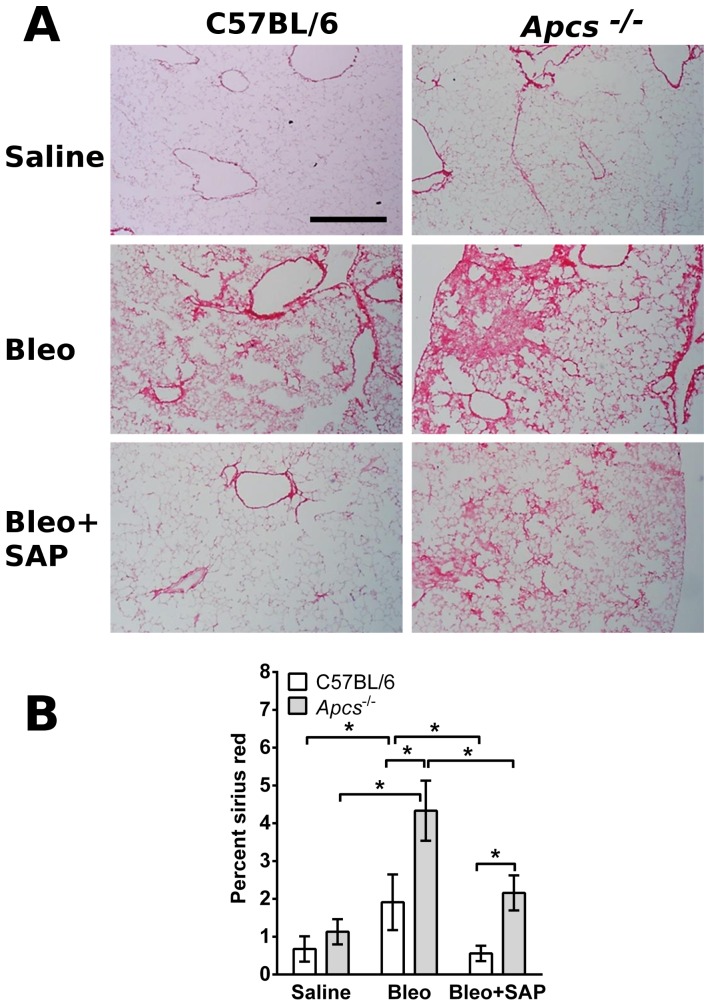
Changes in collagen content in C57BL/6 and *Apcs^-/-^* mice following bleomycin aspiration. **A**) Day 21 lungs were stained with picrosirius red to show collagen deposition. Bar is 500 μm. **B**) The percentage area stained with picrosirius red was quantified as a percentage of the total area of the lung. Significance was determined by ANOVA within treatment groups and t-test when comparing C57BL/6 and *Apcs^-/-^* mice. *p<0.05. (n = 4-6 mice per group).

The data above suggest that *Apcs^-/-^* mice have a more severe inflammatory response and impaired lung function following bleomycin aspiration. To determine if *Apcs^-/-^* mice also have a more severe fibrotic response, lung sections were stained with picrosirius red to detect collagen. Compared to C57BL/6 mice, at 21 days after bleomycin treatment, *Apcs^-/-^* mice had increased numbers and size of fibrotic lesions and more extensive deposition of collagen (Figure 11). In addition, in both C57BL/6 and *Apcs^-/-^* mice, SAP injections significantly reduced picrosirius red staining (Figure 11A and B). These data suggest that a phenotype of *Apcs^-/-^* mice is an increased fibrotic response to bleomycin, that this phenotype can be rescued by SAP injections, and that treatment with exogenous SAP can reduce fibrosis.

## Discussion

We found that compared to C57BL/6 mice, *Apcs^-/-^* mice had a persistent inflammatory response and increased fibrosis in the lungs after bleomycin aspiration. However, the inflammatory cells present during the initial phases of inflammation were similar between C57BL/6 and *Apcs* ^-/-^ mice, suggesting that the initial response to the insult is independent of SAP. These data suggest that a key function of SAP is to promote the resolution of inflammation and fibrosis.

Many groups have shown that SAP injections can reduce inflammation and fibrosis induced by multiple different stimuli [3], [84]. However, in all these experimental systems, endogenous SAP would be present and the stimuli used to induce inflammation and fibrosis may have also elevated endogenous SAP levels. However, the single bleomycin aspiration dose we used did not significantly increase plasma SAP levels. At day 7 following bleomycin aspiration, there was no difference in the number of cells recovered from the BAL of C57BL/6 and *Apcs^-/-^* mice, although we did observe that SAP injections reduced the BAL cell numbers in both strains of mice. These data suggest that the initial inflammatory response to bleomycin is similar in C57BL/6 and *Apcs^-/-^* mice, and that the levels of endogenous SAP are incapable of moderating the inflammatory response. These data may also help explain the strain variation in response to bleomycin. C57BL/6 mice are sensitive to bleomycin-induced lung fibrosis, but Balb/c and DBA/2 strains are resistant [89], [90]. The different strain sensitivities to bleomycin have been associated to a variety of genes including bleomycin hydrolase [58], [91]. However, SAP may also play a crucial role as C57BL/6 mice have low levels of SAP (5–10 μg/ml) whereas the serum concentration of SAP in Balb/c and DBA/2 mice is 50–100 μg/ml [27], [86].

At day 21 following bleomycin aspiration, compared to C57BL/6 mice, *Apcs^-/-^* mice had significantly more cells in the BAL, and these cells contained a significant number of inflammatory CD11b+ macrophages. The cells present in the lung tissue of *Apcs^-/-^* mice also contained increased numbers of CD11b+ inflammatory macrophages, and CD206+ M2a pro-fibrotic macrophages. Compared to the C57BL/6 mice, the overall inflammatory and fibrotic response was elevated and persistent in the *Apcs^-/-^* mice, although the composition of the cells found was not significantly different. These data suggest that the inflammatory response is not aberrant in *Apcs^-/-^* mice, but that the absence of endogenous SAP leads to the persistence of signals that drive inflammation. As SAP binds to apoptotic cells and other debris formed during inflammation, and promotes phagocytosis of this material, the absence of SAP in *Apcs^-/-^* mice, or reduced levels of SAP in susceptible individuals, may be an additional contributing factor to persistent inflammation and fibrosis following inflammation [32], [92].

The observation that there was no significant difference in the BAL protein composition of C57BL/6 and *Apcs^-/-^* mice following bleomycin aspiration suggests that SAP has no protective effect on lung epithelial cells. However, following bleomycin exposure the apoptotic lung epithelial cells, when coated with SAP, are likely to be removed by phagocytosis without generating a pro-inflammatory response [30], [33], [93]–[95]. This agrees with previous data indicating that following TGFβ-induced lung fibrosis, exogenous SAP has no effect on the apoptosis of lung cells during the first week [32]. In addition, we have shown that SAP injections can be delayed for 7–10 days after an inflammatory insult, and yet still reduce inflammation and fibrosis [24], [32].

Compared to C57BL/6 mice, *Apcs^-/-^* spleen cells had an increased numbers of IgG binding cells, altered FcγR expression, and increased inhibition of fibrocyte differentiation by SAP, indicating that endogenous SAP alters the expression of FcγR, and/or binding of SAP to the FcγR. These data also indicate that an additional function of endogenous SAP is to modulate FcγR expression and/or IgG binding, possibly to modulate the inflammatory response.

Compared to C57BL/6 mice, naïve *Apcs^-/-^* mice have significantly smaller lungs, with increased collagen content. This correlates with reduced pulse Ox and increased pulse distension in the *Apcs^-/-^* mice. These data suggest that the lungs of *Apcs^-/-^* mice are less efficient at oxygenating the blood, and that the heart is compensating to overcome this problem. These findings may be related to the reduced number and/or altered functionality of fibrocytes detected in the spleens of *Apcs^-/-^* mice. Together, our results suggest that endogenous and exogenous SAP levels are important in the regulation of bleomycin-induced lung inflammation and fibrosis, and that SAP promotes the resolution of inflammation and reduces fibrosis by multiple mechanisms.
